# Development of an experimental model for vascularized adrenal gland transplantation in rats

**DOI:** 10.3389/fsurg.2025.1749069

**Published:** 2026-01-12

**Authors:** Cumhur Ozcan, Selcuk Mevlut Hazinedaroglu, Tugbay Tug

**Affiliations:** 1 Department of General Surgery, Faculty of Medicine, Mersin University, Mersin, Türkiye; 2 Department of General and Transplantation Surgery, Ankara University, Ankara, Türkiye; 3 Department of General Surgery, Ankara University, Ankara, Türkiye

**Keywords:** adrenal insufficiency, adrenal transplantation, microsurgical technique, rat experimental model, vascularized graft model

## Abstract

**Aim:**

The adrenal gland is a vital endocrine organ responsible for maintaining physiological homeostasis. Bilateral adrenalectomy results in adrenal insufficiency, requiring lifelong hormone replacement therapy. This study aimed to establish a reproducible experimental model for vascularized adrenal gland transplantation in rats.

**Materials and methods:**

Twenty male Wistar Albino rats (180–220 g) were randomly assigned into two equal groups (*n* = 10 each): control and transplantation. The control group underwent bilateral total adrenalectomy. In the transplantation group, vascularized adrenal grafts—including the adrenal artery and vein with attached aortic and inferior vena cava segments—were anastomosed end-to-side to the recipient's femoral artery and vein using 10-0 nylon sutures under general anesthesia. On postoperative day 15, all recipients underwent bilateral adrenalectomy. On day 100, animals were sacrificed for macroscopic and histopathological evaluation of graft viability.

**Results:**

All control rats died within 15 days, whereas all transplanted rats survived throughout the 100-day observation period. Macroscopic inspection revealed viable grafts without vascular compromise. Histopathological analysis demonstrated preserved cortical and medullary architecture, confirming the long-term viability of the transplanted adrenal glands.

**Conclusion:**

This study presents a technically feasible and reproducible model for vascularized adrenal gland transplantation in rats. The model provides a reliable experimental platform for future research on adrenal physiology and transplantation surgery.

## Introduction

Adrenal insufficiency is a rare but potentially life-threatening endocrine disorder characterized by impaired production of glucocorticoids, and in some cases mineralocorticoids. Recent epidemiological data estimate an incidence of approximately 93–110 cases per million individuals, with a persistent risk of adrenal crisis despite treatment (1, 2). Autoimmune adrenal destruction, infections such as tuberculosis, malignancy, hemorrhage, and ischemia represent the most common etiologies (3). Lifelong glucocorticoid and mineralocorticoid replacement remains the standard therapy for patients with primary adrenal failure or those undergoing bilateral adrenalectomy; however, exogenous steroid administration fails to mimic the physiological circadian rhythm of cortisol secretion and may require dose modifications during stress, illness, or trauma (4, 5). As a result, patients often experience impaired stress response, metabolic instability, reduced quality of life, and ongoing risk of adrenal crisis (6).

Adrenal transplantation has been explored as a potential alternative to lifelong hormonal therapy by aiming to restore endogenous adrenal function. Although early attempts focused primarily on non-vascularized adrenal autografts, ischemia-related graft loss and inconsistent outcomes limited their clinical utility (7, 8). In recent years, advances in microsurgical techniques and interest in endocrine organ transplantation have renewed attention toward vascularized adrenal grafts, which demonstrate improved perfusion, cellular viability, and potential for physiological hormone secretion (9–11). Furthermore, emerging regenerative strategies—including adrenocortical cell implantation, stem-cell derived adrenal organoids, and bioengineered adrenal tissue—have emphasized the need for reliable *in vivo* models to investigate adrenal physiology and graft function (12–14).

Despite this progress, there remains a lack of standardized and reproducible small-animal models for vascularized adrenal transplantation. Establishing such a model would provide an essential platform for evaluating graft viability, endocrine recovery, and ischemia-reperfusion mechanisms, while supporting future translational research in adrenal replacement therapy. Previous vascularized adrenal transplantation models have been limited by technically demanding intraperitoneal dissection, inconsistent graft perfusion, and a lack of long-term reproducible outcomes. Therefore, a simplified and standardized small-animal model with reliable vascular perfusion and durable graft survival is still needed. Our study aims to address this gap by introducing a reproducible femoral-based vascularized adrenal transplantation model.

The present study aimed to develop a technically feasible and reproducible rat model of vascularized adrenal gland transplantation and to assess long-term graft viability using survival and histopathological outcomes.

## Materials and methods

### Animals

This study was conducted at the Ankara University Experimental Research Laboratory and approved by the XX Ethics Committee for Animal Experiments (Approval No: 102-2673, Date: 20 November 2006). All procedures adhered to the National Institutes of Health Guidelines for the Care and Use of Laboratory Animals and followed the ARRIVE guidelines.

A total of 30 male Wistar Albino rats (180–220 g) were housed under controlled environmental conditions (22 ± 2 °C, 60% ± 5% humidity, 12/12 h light–dark cycle) with *ad libitum* access to standard pellet chow and water. Rats were acclimatized for one week prior to surgery and monitored daily for general condition, hydration, and activity.

Animals were randomly allocated into two groups (*n* = 10 per group). An additional 10 rats served as donors for graft procurement. The sample size (*n* = 10 per group) was chosen based on feasibility constraints and consistency with previously published small-animal microsurgical transplantation models. The control group underwent bilateral total adrenalectomy, whereas the transplantation group underwent vascularized adrenal gland transplantation followed by bilateral adrenalectomy on postoperative day 15.

### Anesthesia and perioperative care

All surgical procedures were performed under general anesthesia using intramuscular ketamine hydrochloride (50 mg/kg) combined with xylazine hydrochloride (10 mg/kg). Adequate anesthesia depth was confirmed by loss of pedal withdrawal reflex and lack of response to tail pinch. No inhalational anesthetics were used. Body temperature was maintained throughout surgery using a thermostatically controlled heating platform (37 °C).

Prophylactic antibiotic therapy consisted of cefazolin sodium (20 mg/kg IM) administered 30 min before surgery. Hydration was supported with 10 mL/kg of sterile saline administered subcutaneously at the start of the operation. Postoperative analgesia was provided with buprenorphine (0.05 mg/kg SC every 12 h for 48 h). Animals were monitored daily for wound integrity, hydration, and overall health.

### Operation of the control group

**Figure 1:**
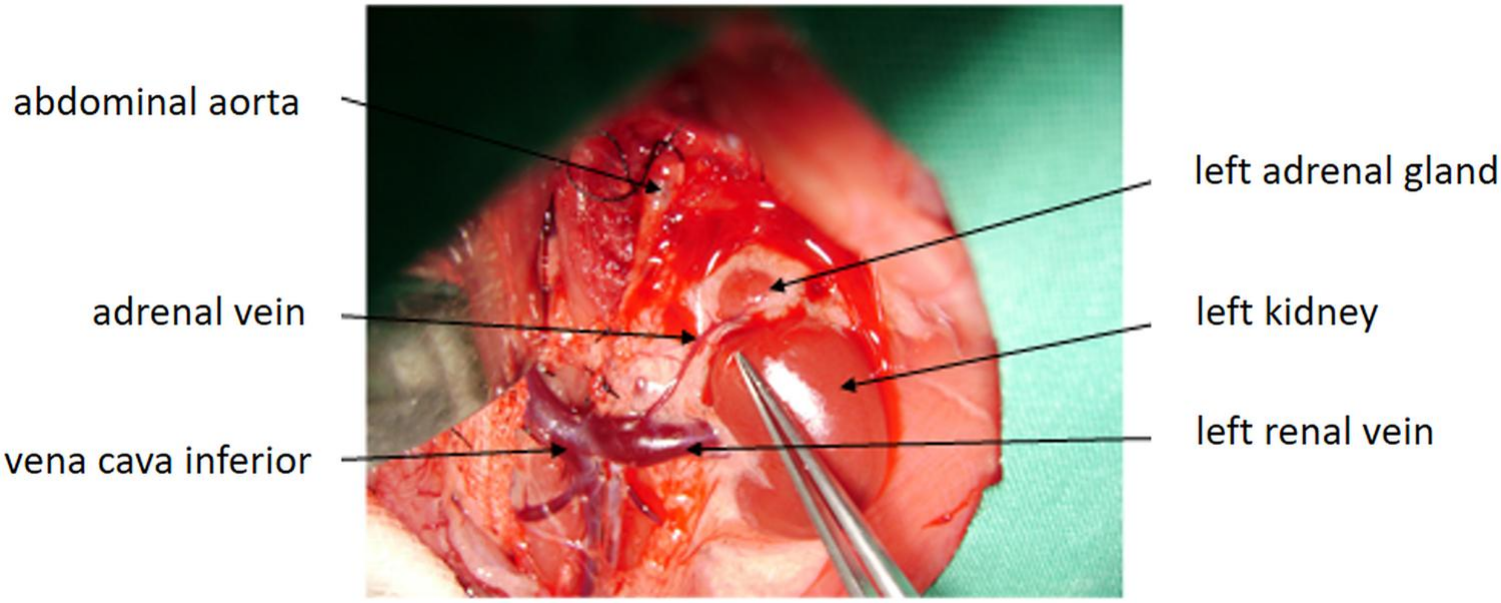
Anatomical exposure of the left adrenal gland and surrounding vascular structures. The abdominal aorta, inferior vena cava, left renal vein, left adrenal vein, left kidney, and left adrenal gland are clearly visualized.

Under general anesthesia, a midline laparotomy was performed. The small and large intestines were gently retracted to the right, and the stomach was elevated to expose the retroperitoneum (Figure 1). The left adrenal gland was identified and dissected free. The adrenal artery and vein were ligated using 5-0 silk sutures, and the gland was excised. An identical procedure was performed on the right adrenal gland. The abdominal fascia and skin were closed separately with 4-0 nylon sutures.

### Donor operation and graft preparation

Following sterile preparation, a midline abdominal incision was made. The stomach, intestines, left hepatic lobe, and spleen were gently retracted to the right to expose the left suprarenal region. The left adrenal gland and upper pole of the left kidney were visualized clearly. Warm sterile saline was intermittently applied to prevent tissue desiccation.

The abdominal aorta and inferior vena cava (IVC) were exposed by blunt dissection, and all lateral branches were ligated using 7-0 or 8-0 silk sutures. The aorta was dissected proximally above the origin of the left adrenal artery and distally to the aortic bifurcation. Both ends were suspended with 6-0 silk to facilitate cannulation. A 21-gauge angiocatheter was inserted into the infrarenal aorta.

**Figure 2:**
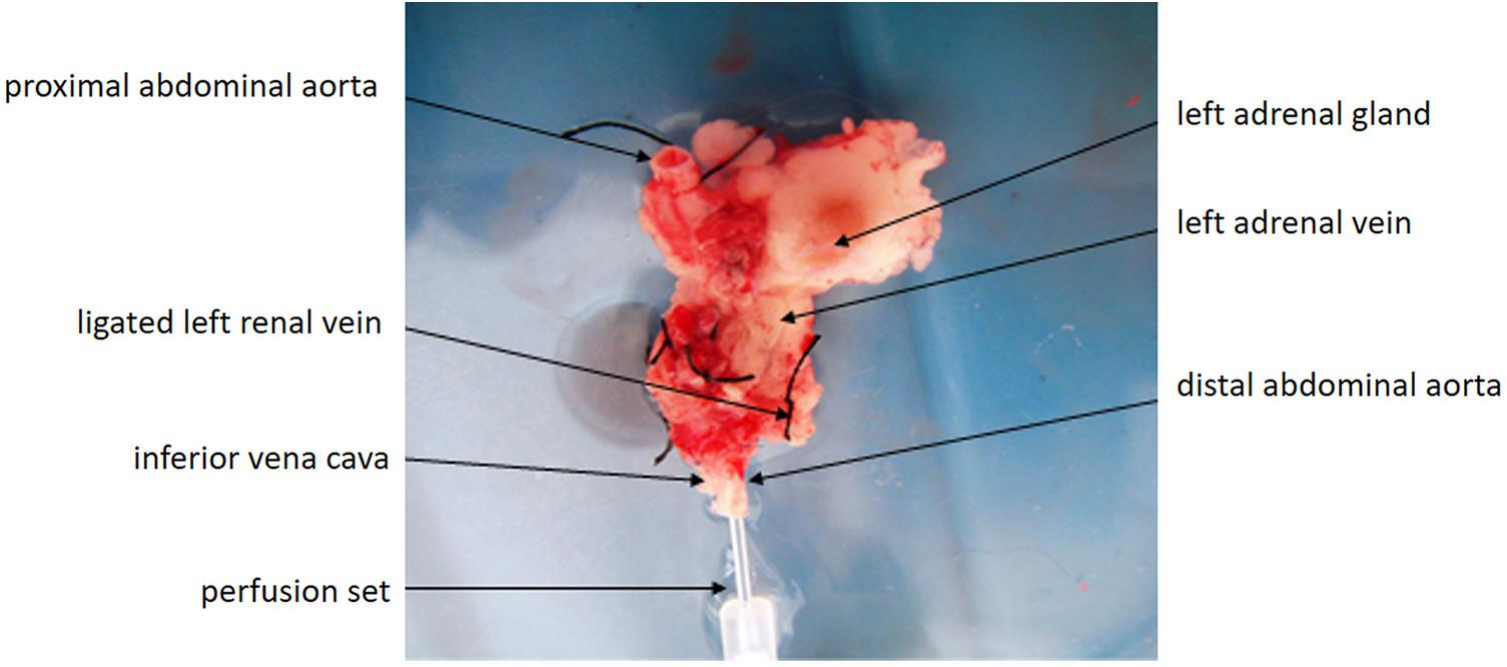
Excised vascularized adrenal graft preserved with attached segments of the abdominal aorta and inferior vena cava following *in-situ* cold perfusion. Perfusion was performed using 4°C heparinized lactated Ringer's solution until blanching of the adrenal gland was achieved.

The left renal artery and vein were ligated distal to the entry of the adrenal vein, and a left nephrectomy was performed. Cold (4 °C) heparinized lactated Ringer's solution was used for *in situ* perfusion until the gland appeared blanched (Figure 2). The adrenal gland, along with its vascular pedicle and attached segments of aorta and IVC, was excised *en bloc* and stored in cold preservation solution until transplantation.

### Operation of the transplantation (recipient) group

**Figure 3:**
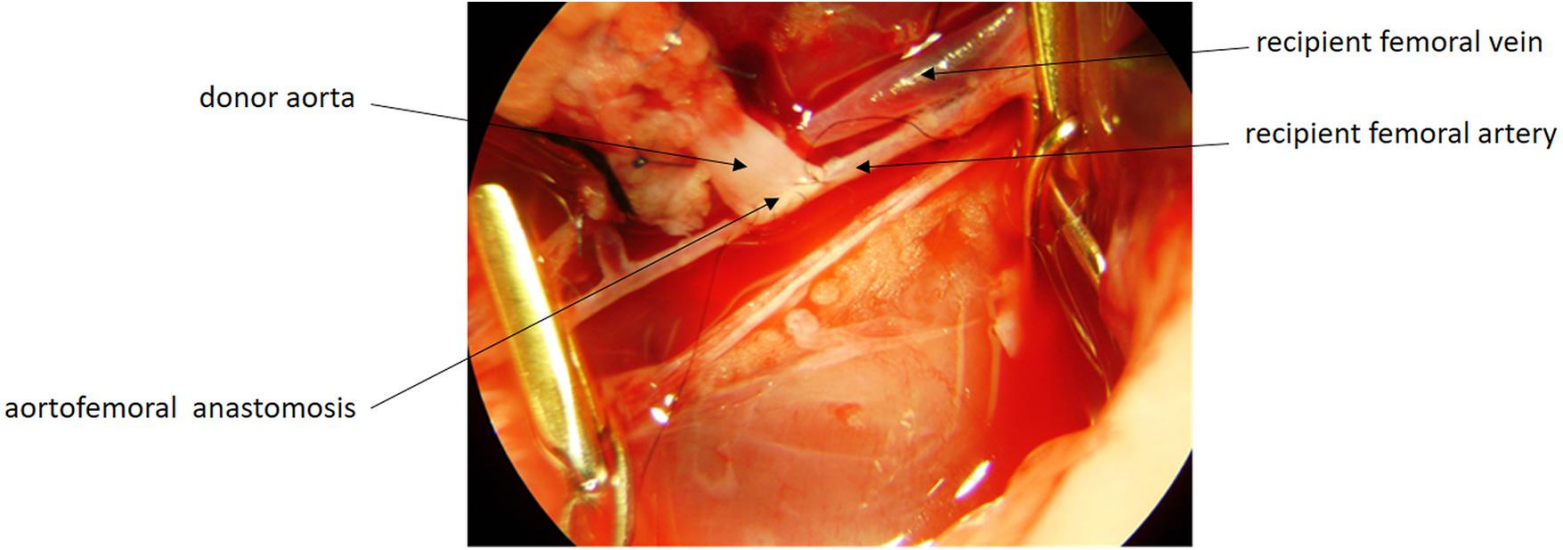
Microsurgical end-to-side anastomosis between the donor aortic segment and the recipient femoral artery using interrupted 10-0 nylon sutures. Adequate perfusion was verified by immediate color change of the graft after completion of the anastomosis.

Recipient rats were anesthetized as described previously. The femoral artery and vein were selected as the recipient vessels because they allow superficial access without laparotomy, provide easier microsurgical handling, minimize postoperative adhesions, and facilitate reliable monitoring and retrieval of the graft. A left inguinal incision was made, and the femoral neurovascular bundle was identified. The femoral artery and vein were isolated, and end-to-side microvascular anastomoses were performed between the donor aortic segment and the recipient femoral artery, and between the donor IVC segment and the recipient femoral vein, using interrupted 10-0 nylon sutures (Figure 3). Reperfusion was confirmed by the immediate color change of the adrenal gland and the visible filling of the adrenal vein following completion of the microvascular anastomoses. All assessments of graft perfusion, thrombosis, or vascular leakage were performed solely by macroscopic inspection, as Doppler ultrasonography suitable for small-animal intraoperative evaluation was not available. The incision was closed in two layers. The mean duration of the transplantation procedure was approximately 60–75 min, including graft preparation, vascular isolation, and microvascular anastomoses. The estimated warm ischemia time during graft handling and anastomosis was approximately 6–10 min. No intraoperative complications such as bleeding, vasospasm, or anastomotic leakage occurred in any of the animals.

### Euthanasia

On postoperative day 100, all animals were euthanized under deep anesthesia induced by an intraperitoneal overdose of ketamine hydrochloride (200 mg/kg). Complete cessation of cardiac and respiratory activity for ≥5 min was used to confirm death, in accordance with the AVMA Guidelines for the Euthanasia of Animals (2020).

### Histopathological evaluation

The transplanted adrenal glands were harvested *en bloc* with their surrounding tissues to preserve orientation. The specimens were immediately fixed in 10% neutral-buffered formalin for 24–48 h and subsequently processed for paraffin embedding. Serial sections (5 µm thick) were obtained and stained with hematoxylin and eosin (H&E). Microscopic examinations were performed using a light microscope at ×40 and ×100 magnifications by an experienced pathologist blinded to the experimental groups.

Histopathological evaluation included assessment of cortical and medullary architecture, the presence or absence of necrosis, fibrosis, hemorrhage, congestion, and cellular atrophy. The integrity of the adrenal capsule and vascular structures was also examined to determine overall graft viability and tissue preservation.

### Statistical analysis

This study was designed as a descriptive experimental model. Due to the small sample size and the qualitative nature of the data, no formal statistical comparisons were performed. Survival outcomes and histopathological findings were presented descriptively to demonstrate model feasibility and graft viability. In accordance with the descriptive nature of the study, no hypothesis-driven statistical comparison was performed. A Kaplan–Meier survival curve was generated to visually present survival differences between groups.

## Results

All rats in the control group died within 15 days following bilateral adrenalectomy, whereas all animals in the transplantation group survived throughout the 100-day observation period. No graft loss, anastomotic leakage, or thrombotic complications were encountered.

Macroscopic evaluation on postoperative day 100 revealed well-perfused adrenal grafts with intact vascular pedicles and normal reddish-brown coloration. There were no signs of thrombosis, necrosis, fibrosis, or vascular leakage at the anastomotic sites.

Throughout the postoperative follow-up, transplanted rats demonstrated normal activity, grooming behavior, hydration, and weight stability, with no signs of adrenal insufficiency. In contrast, control animals progressively developed lethargy, dehydration, and weight loss before mortality.

**Figure 4:**
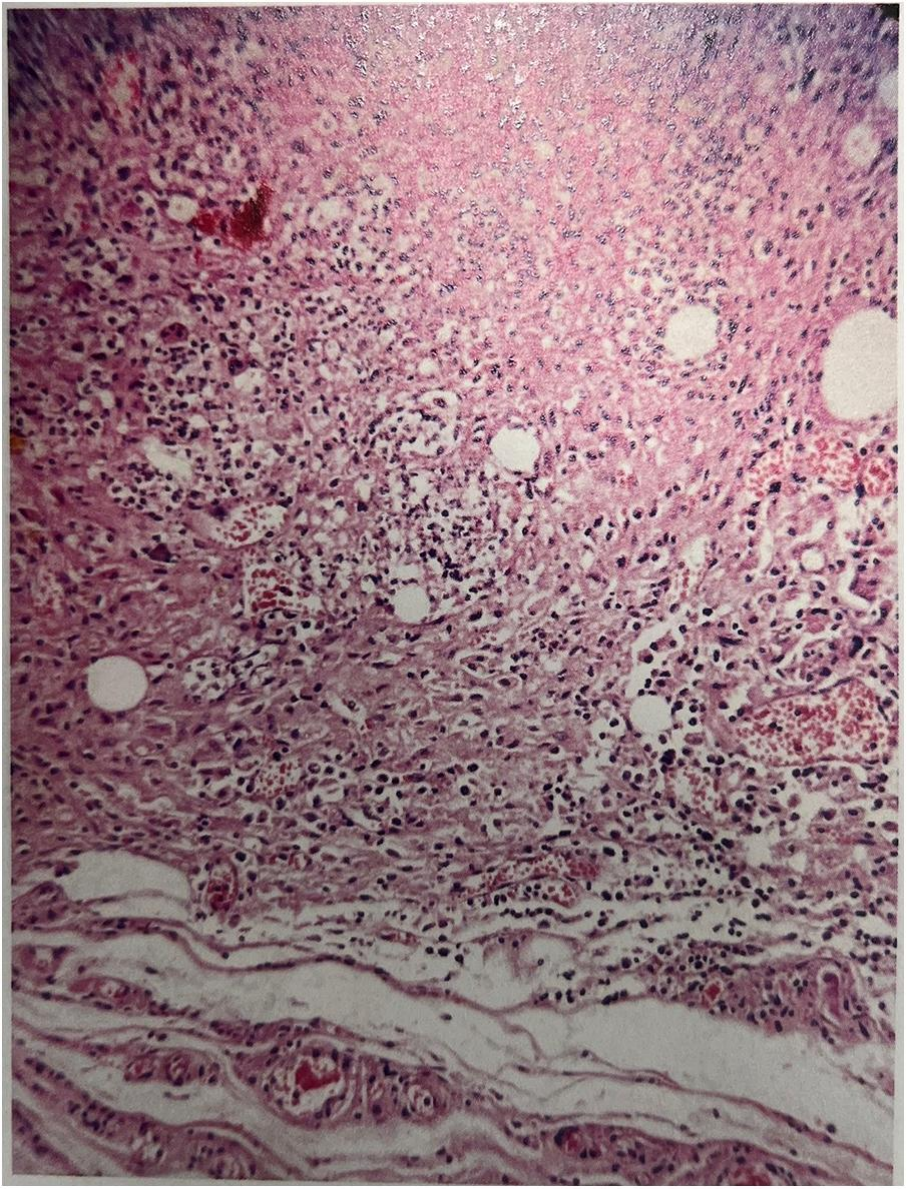
Histological section of the transplanted adrenal gland on postoperative day 100 showing preserved cortical and medullary architecture (H&E stain, ×40). The zona glomerulosa, zona fasciculata, and zona reticularis remain distinguishable, indicating long-term structural viability of the graft.

Histopathological examination of paraffin-embedded, hematoxylin–eosin-stained sections demonstrated well-preserved cortical and medullary architecture in all transplanted grafts (Figure 4). The zona glomerulosa, zona fasciculata, and zona reticularis were clearly distinguishable, confirming preservation of normal adrenal structure. No necrosis, fibrosis, or hemorrhage was observed. Mild vascular congestion and focal cellular atrophy were noted in a few samples but did not distort the overall tissue architecture.

Each histopathologic feature—including necrosis, fibrosis, hemorrhage, cortical atrophy, and structural integrity—was semi-quantitatively graded on a 0–3 scale (0 = none, 1 = mild, 2 = moderate, 3 = severe). All grafts scored 0 for necrosis and fibrosis, and ≤1 for congestion and atrophy, confirming excellent graft viability and histological preservation 100 days after transplantation.

## Discussion

Adrenal insufficiency remains a challenging endocrine disorder, particularly in patients who require bilateral adrenalectomy, since lifelong glucocorticoid and mineralocorticoid replacement therapy fails to fully reproduce physiological cortisol rhythms and may lead to metabolic instability, impaired stress response, and reduced quality of life (1–3). For this reason, adrenal transplantation has long been explored as a potential strategy to restore endogenous steroidogenesis and minimize the burden of chronic hormone replacement.

Early attempts at adrenal autotransplantation date back to the seminal work of Franksson in 1959 (4). Subsequent clinical and experimental studies investigated various implantation sites, including subcutaneous, intraperitoneal, and orthotopic locations, reporting success rates that generally ranged between 30% and 40% (5–9). However, these early approaches were largely limited by inadequate revascularization and ischemic graft necrosis, which underscored the need for techniques involving vascularized grafts.

To overcome these limitations, vascularized adrenal graft models were developed. Xu et al. (10) demonstrated the feasibility of adrenal autotransplantation with preserved vascular inflow and outflow, while Liu et al. (11) later introduced a microsurgical model that utilized aortic and inferior vena caval segments to maintain optimal perfusion. More recently, advancements in microsurgical techniques, together with regenerative approaches such as adrenal organoid systems and stem-cell–derived adrenocortical constructs, have renewed interest in standardized and reproducible animal models (12–14).

Previous vascularized adrenal transplantation models have reported variable graft viability and technical complexity. Xu et al. (8) described a model requiring extensive intraperitoneal dissection with limited long-term survival, while Liu et al. (9) introduced aortic and caval pedicles but reported inconsistent graft perfusion. More recently, Kim et al. (10) demonstrated improved microsurgical outcomes but provided only short-term follow-up without standardized reproducibility. Compared with these models, the present study offers a simplified extracavitary approach using femoral vessels, avoids abdominal adhesions, and demonstrates consistent vascular perfusion with preserved adrenal architecture up to 100 days.

In the present study, we developed a simplified and reproducible vascularized adrenal transplantation model in rats using end-to-side anastomosis of donor aortic and IVC segments to the recipient femoral artery and vein. This technique eliminated the need for laparotomy, minimized postoperative adhesions, and facilitated both clinical monitoring and graft retrieval. The 100% survival rate and histopathological confirmation of preserved cortical and medullary architecture at 100 days indicate durable graft viability and adequate revascularization, findings that are consistent with previously reported vascularized graft models (10–13).

Histological assessment revealed no necrosis, fibrosis, or hemorrhage, with only mild vascular congestion and focal atrophy in a few sections—findings that confirm preserved functional morphology. These results reinforce the concept that vascularized grafts provide superior viability and structural integrity compared to non-vascularized cortical implants, emphasizing the physiological advantage of restoring the native adrenal blood supply.

The primary limitation of this study is the absence of biochemical evaluation of adrenal hormonal activity. Although survival and histological findings strongly support successful graft function, future investigations should include measurements of serum corticosterone and aldosterone levels, extended follow-up periods, and assessment of immunological responses to enhance translational relevance. In addition, the relatively small sample size and the use of a syngeneic rat model—which eliminates rejection mechanisms—limit the direct extrapolation of these findings to human transplantation. Incorporation of endocrine assays, immunological analysis, and functional stimulation tests will be essential to validate the model as a foundation for future therapeutic studies. Moreover, the use of a syngeneic rat model eliminates immunological rejection responses and does not fully replicate the anatomical and physiological characteristics of human adrenal transplantation, which limits the direct clinical translatability of these findings.

From a translational perspective, adrenal transplantation presents several ethical and practical challenges compared with lifelong glucocorticoid and mineralocorticoid replacement therapy, which remains the clinical standard for adrenal insufficiency. Although vascularized adrenal grafts may theoretically restore physiological steroidogenesis and eliminate the need for stress-dose adjustments, the requirement for immunosuppression, donor availability, procedural complexity, and long-term safety concerns currently limit any clinical application. Therefore, the present model should be viewed as a platform for experimental research rather than a therapy intended for near-term clinical translation.

In conclusion, the vascularized adrenal transplantation model described herein is technically feasible, reproducible, and demonstrates excellent long-term graft viability. This model provides a reliable experimental platform for further studies investigating adrenal physiology, ischemia–reperfusion injury, regenerative approaches, and potential clinical applications of vascularized adrenal transplantation.

## Conclusion

This study demonstrated that the proposed vascularized adrenal gland transplantation model in rats is technically feasible, reproducible, and associated with excellent long-term survival. The preservation of cortical and medullary architecture at 100 days confirmed successful revascularization and sustained graft viability. This experimental model provides a reliable and practical platform for future investigations into adrenal physiology, ischemia–reperfusion injury, and potential advancements in adrenal transplantation surgery.

## Data Availability

The original contributions presented in the study are included in the article/supplementary material, further inquiries can be directed to the corresponding author/s.

## Supplementary material

Supplementary Figure 1 Kaplan–Meier survival curves of the control and transplantation groups. The control group (*n* = 10) showed complete mortality within the first 15 postoperative days following bilateral adrenalectomy. In contrast, the transplantation group (*n* = 10) demonstrated 100% survival throughout the 100-day observation period after vascularized adrenal gland transplantation. The curve illustrates the marked survival advantage conferred by successful graft perfusion and preservation.
