# Supplementary figures and images for: Development of an experimental model for vascularized adrenal gland transplantation in rats
Source: Front Surg. 2026 Jan 12;12:1749069. doi: 10.3389/fsurg.2025.1749069 (PMC12833046; doi:10.3389/fsurg.2025.1749069)

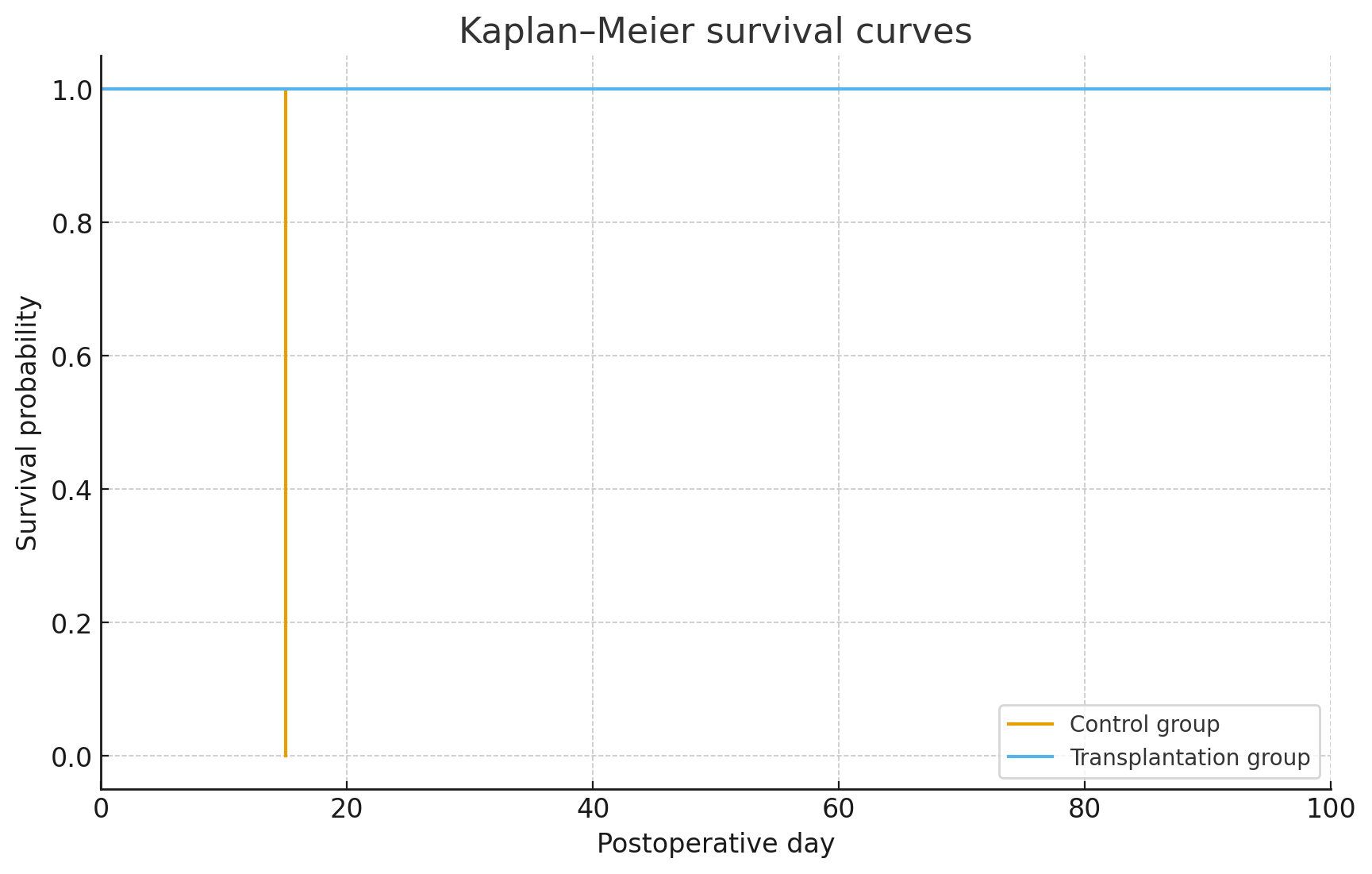

Supplement: Supplementary Figure 1 — Kaplan–Meier survival curves of the control and transplantation groups. The control group (n = 10) showed complete mortality within the first 15 postoperative days following bilateral adrenalectomy. In contrast, the transplantation group (n = 10) demonstrated 100% survival throughout the 100-day observation period after vascularized adrenal gland transplantation. The curve illustrates the marked survival advantage conferred by successful graft perfusion and preservation. [file Image1.png]
